# USP14: Structure, Function, and Target Inhibition

**DOI:** 10.3389/fphar.2021.801328

**Published:** 2022-01-05

**Authors:** Feng Wang, Shuo Ning, Beiming Yu, Yanfeng Wang

**Affiliations:** Key Laboratory of Molecular Medicine and Biotherapy, School of Life Science, Beijing Institute of Technology, Beijing, China

**Keywords:** ubiquitin-specific protease 14, structure, regulation, pathophysiological function, signaling pathway, target inhibition, disease

## Abstract

Ubiquitin-specific protease 14 (USP14), a deubiquitinating enzyme (DUB), is associated with proteasomes and exerts a dual function in regulating protein degradation. USP14 protects protein substrates from degradation by removing ubiquitin chains from proteasome-bound substrates, whereas promotes protein degradation by activating the proteasome. Increasing evidence have shown that USP14 is involved in several canonical signaling pathways, correlating with cancer, neurodegenerative diseases, autophagy, immune responses, and viral infections. The activity of USP14 is tightly regulated to ensure its function in various cellular processes. Structural studies have demonstrated that free USP14 exists in an autoinhibited state with two surface loops, BL1 and BL2, partially hovering above and blocking the active site cleft binding to the C-terminus of ubiquitin. Hence, both proteasome-bound and phosphorylated forms of USP14 require the induction of conformational changes in the BL2 loop to activate its deubiquitinating function. Due to its intriguing roles in the stabilization of disease-causing proteins and oncology targets, USP14 has garnered widespread interest as a therapeutic target. In recent years, significant progress has been made on identifying inhibitors targeting USP14, despite the complexity and challenges in improving their selectivity and affinity for USP14. In particular, the crystal structures of USP14 complexed with IU1-series inhibitors revealed the underlying allosteric regulatory mechanism and enabled the further design of potent inhibitors. In this review, we summarize the current knowledge regarding the structure, regulation, pathophysiological function, and selective inhibition of USP14, including disease associations and inhibitor development.

## Introduction

Ubiquitination is an essential posttranslational modification process in both prokaryotic and eukaryotic cells, whereby substrate protein is covalently attached to ubiquitin through isopeptide bonds catalyzed by the E1-E2-E3 ligase cascade to be marked for degradation (Schulman and Harper, 2009; Ye and Rape, 2009; Buetow and Huang, 2016). This modification coordinates gene transcription, DNA damage repair, and the cell cycle, and has been found to be linked to a majority of cancer-related pathways (Reyes-Turcu et al., 2009; Sacco et al., 2010; Komander and Rape, 2012). Different chain linkages, such as linkage of K6, K11, K27, K29, K33, K48, and K63-linked linear ubiquitin chain dictate different cellular fates. For example, linear or K63 linkages belong to the nondegrading polyubiquitin chains that promote protein complex formation, whereas K11 or K48-linked ubiquitin chains promote protein degradation (Komander and Rape, 2012). Conversely, deubiquitinating enzymes (DUBs) are proteases that bind to ubiquitin-tagged substrates or polyubiquitin chains, hydrolyzing the isopeptide bond between ubiquitin and the lysine side chain or N-terminal methionine of linear polyubiquitin chains. These two types of enzymes function in combination to accurately regulate the dynamic balance of protein ubiquitination (Dikic, 2017; Clague et al., 2019).

At present, more than 100 DUBs have been discovered in humans and are classified into six distinct families according to their structure and function: ubiquitin-specific proteases (USPs), ubiquitin C-terminal hydrolases (UCHs), Machado-Joseph domain-containing proteases, ovarian tumor proteases, motif-interacting with ubiquitin containing proteases, and JAMM/MPN domain-associated Zn-dependent metalloproteases (Nijman et al., 2005; Mevissen and Komander, 2017). The USPs family is the most frequently studied DUB family and is comprised of more than 60 members.

USP14, belonging to the USPs family, is known to be extensively engaged in varying canonical cellular signaling pathways, including nuclear factor kappa B (NF-κB) and Wnt/β catenin signaling pathways (Harrigan et al., 2018; Li et al., 2021). Studies have demonstrated that the activity of USP14 is strictly regulated through its association with proteasome and several kinds of postmodifications, such as phosphorylation (Xu et al., 2015; Wang and Wang, 2021b). Dysregulation of USP14 causes pathological conditions, such as cancer, neurodegenerative disease, autophagy, immune responses, and viral infections (Wang et al., 2021a). Identifying inhibitors targeting USP14 has therefore become an active arena of research for both research institutions and pharmaceutical companies. Over the past years and despite the complexity and challenges in improving the selectivity and affinity of inhibitors targeting USP14 a lot of progress has been made in this field (Harrigan et al., 2018). Recently, our team determined the crystal structure of USP14 in complex with IU1-series inhibitors, designed a more potent inhibitor (IU1-248), and discovered the allosteric regulatory mechanism underlying the activity and selectivity of USP14 inhibitors (Wang et al., 2018). To this day, approximately 40 USP14 inhibitors have been reported, although most are weak and multitargeted agents.

In this review, we attempted to summarize the structure, function, and regulation of USP14 in cellular and physiological processes, as well as report the recent findings regarding its role in the progression of various diseases and development of inhibitors.

## Structure of USP14

**FIGURE 1:**
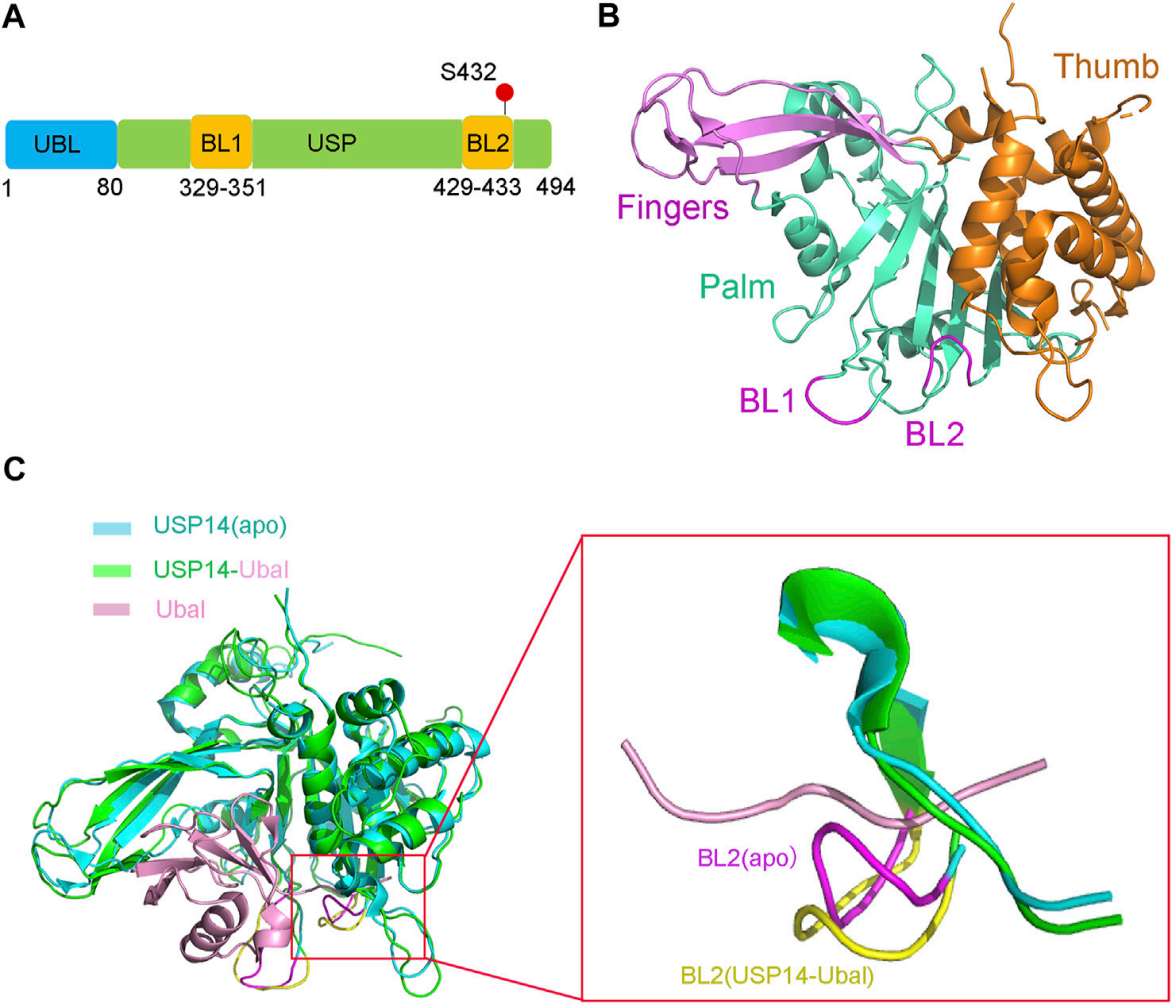
Structure of USP14 in the inactive and active conformation with Ubal. **(A)** Domain structure and modification of the full-length USP14. The full-length human USP14 have 494 residues including N-terminal Ubl domain and C-terminal catalytic domain. BL1 and BL2 in USP domain are key to the activity of USP14. The phosphorylation site of USP14 is labeled in red color. **(B)** The inactive crystal structure of USP14 including Fingers, Palm and Thumb domains is colored in magnet, cyan and orange color, respectively. The conformation of BL1 and BL2 are colored in magnet. **(C)** Crystal structure of USP14 in complexed with Ubal. Left: Compared the structure of USP14 between USP14(apo) and USP14-Ubal. USP14 in the free and complex are shown in cyan and green, respectively. The Ubal is shown in pink. Right: Compared the BL2 between USP14(apo) and USP14-Ubal(complex). USP14 in the free and complex are shown in cyan and green, respectively. BL2 in the free and complex are shown in magnet and yellow, respectively. The Ubal is shown in pink.

The full-length human USP14 contains 494 amino acids, which comprise two structural domains according to their function, a N-terminal ubiquitin-like (UBL) domain and a C-terminal catalytic USP domain (residues 96–494) (Hu et al., 2005) (Figure 1A). The N-terminal UBL domain of USP14 is an important regulator of proteasomal activity, whereas the C-terminal USP domain is responsible for its deubiquitinating enzymatic activity (Shi et al., 2016; Kim and Goldberg, 2018b).

The USP14 catalytic domain displays a classic structure similar to most USPs, resembling an extended right hand with three subdomains, a finger, palm, and thumb (Figure 1B), which constitute the ubiquitin binding cleft. The finger subdomain consists of five β strands, including β2–β4, β6, and β7. The palm subdomain contains a 6-strand β sheet (β5, β8, and β10–β13), one short β9 strand, and several surface loops. Two surface loops, named BL1 (residues 329–351) and BL2 (residues 429–433), partially hover above the active site cleft and block the binding of the C-terminus of ubiquitin. The thumb subdomain comprises a six α-helix (α1–α6) strand and one short β strand (β1) (Hu et al., 2005).

Structural comparison between the apo USP14 catalytic domain and USP14-Ubal binary complex revealed that the two surface loops (BL1 and BL2) in the palm subdomain, which are positioned above the catalytic cleft of apo USP14, undergo considerable conformational changes and widen the binding cleft, in order to accommodate the C-terminus of ubiquitin (Figure 1C) (Hu et al., 2005). The side chain of Phe331 on the BL1 loop and the hydrogen bond formed between the side chain of Ser431 on the BL2 loop and Asp199 on the switching loop were found to sterically occlude the catalytic cleft of apo USP14, indicating that the proteasomal-free form of USP14 stays in an autoinhibited form, thus harboring a low level of deubiquitinase activity (Hu et al., 2005).

## Regulation of USP14 Activity

### Proteasome-Dependent Regulation of USP14 Activity

The ubiquitin-proteasome system (UPS) plays essential roles in maintaining homeostasis, ranging from the degradation of unwanted proteins to the regulation of nearly all cellular events in eukaryotic cells (Komander and Rape., 2012; Amm et al., 2014; Gong et al., 2016). In mammalian cells, the 26S proteasome, which is tightly regulated, is mainly responsible for degrading ubiquitin-modified protein substrates (Rock et al., 1994; Leggett et al., 2002; Rock et al., 2014). Trimming of ubiquitin chains on substrates by proteasome-associated DUBs, including Rpn11, Uch37, and USP14 is regarded a crucial regulatory step for UPS (Borodovsky et al., 2001; Verma et al., 2002; Yao and Cohen, 2002; Koulich et al., 2008; Lee et al., 2011). In the case of USP14, it is a major regulator of proteasome, at which it reversibly binds and negatively modulates its activity by deubiquitinating the K48 ubiquitin chains on protein substrates (Borodovsky et al., 2001; Lee et al., 2010).

**FIGURE 2:**
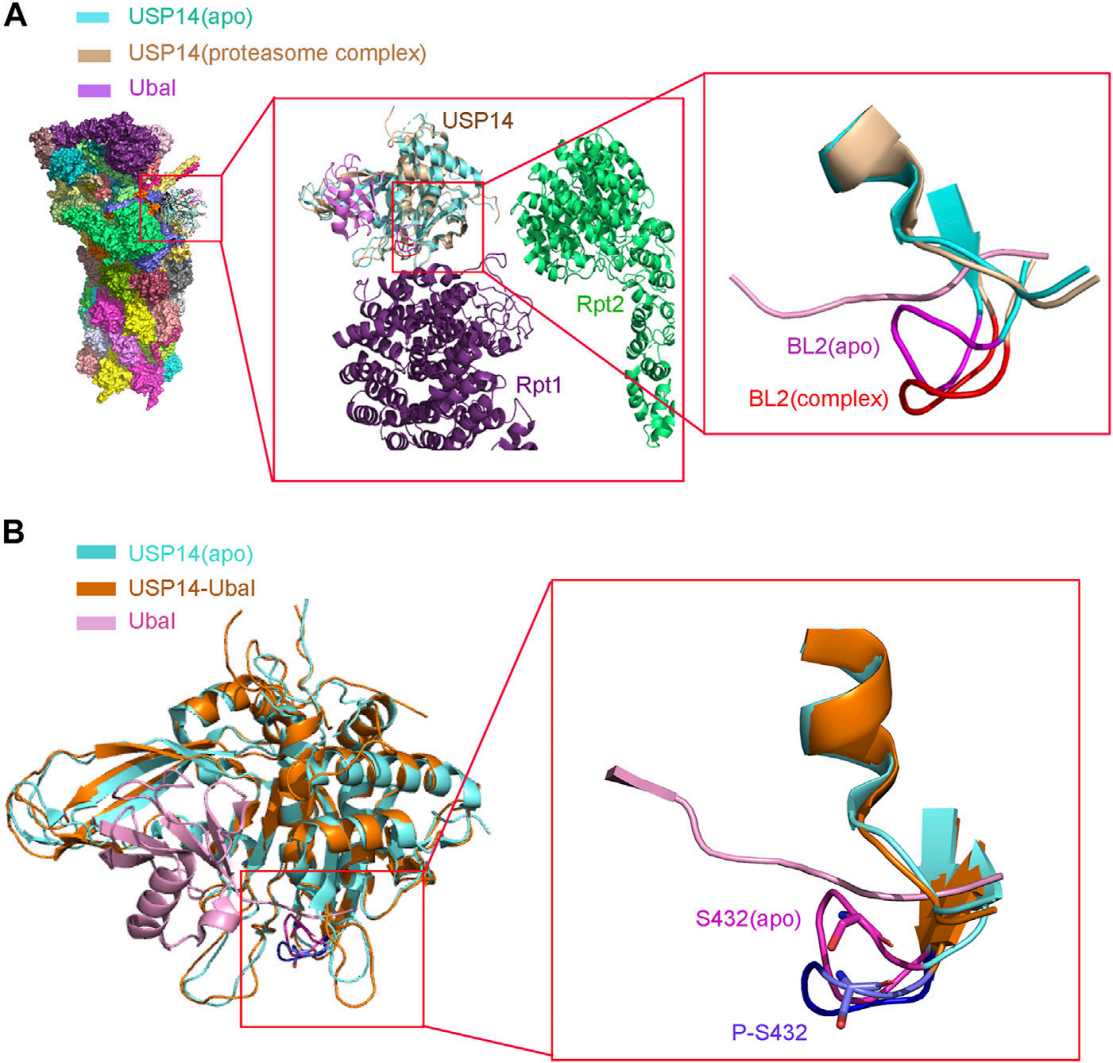
Structure of USP14 catalytic domain regulated by proteasome and phosphorylation. **(A)** Left: Compared the structure of USP14 between USP14(apo) and 26s proteasome-USP14-Ubal(complex). Middle: Enlarged structure of proteasome-USP14-Ubal. Right: Compared the BL2 between USP14(apo) and 26s proteasome-USP14-Ubal (complex). USP14 in the free and complex are shown in cyan and wheat, respectively. BL2 in the free and complex are shown in magnet and red, respectively. The Ubal is shown in pink. **(B)** Left: The structure of USP14(apo) and USP14 in complex with Ubal. Right: Compared the BL2 between USP14(apo) P-S432-USP14. Phosphorylation of USP14 at S432 lead similar conformation changes to USP14-Ubal. BL2 in the free and complex are shown in magnet and purple, respectively.

Purified recombinant USP14, which is in an autoinhibited state *in vitro*, has lower deubiquitinating activity (Hu et al., 2005). However, it is highly activated when associated with proteasome (Lee et al., 2010). A structural study demonstrated that USP14 undergoes a drastic conformational change with the translocation of its two surface loops (BL1 and BL2) upon its association with proteasome, thus allowing the access of the ubiquitin C-terminus to the catalytic active site, in consistency with the structure of the USP14-Ubal complex (Figure 2A) (Aufderheide et al., 2015; Huang et al., 2016). The 26S proteasome-activated USP14 is capable of cleaving single ubiquitin from its substrates and removing the en-bloc ubiquitin chains from substrates ubiquitinated at multiple sites, until only a single chain remains (Lee et al., 2016).

Interestingly, different binding patterns to USP14 direct different forms of proteasomal activity. Studies have shown that the binding of either full-length USP14 or its UBL domain alone represses multiple proteasomal activities through its DUB activity in an allosteric mechanism, whereas USP14 promotes the proteasomal degradation of ubiquitin-conjugated substrates once bound to a ubiquitin chain (Crosas et al., 2006; Hanna et al., 2006; Kim and Goldberg, 2017; Kim and Goldberg, 2018b). A recent cryoEM study of the USP14 yeast homolog Ubp6 revealed that the C-terminal USP domain bound to Ubal or Ub chains relocates near the ATPase ring of proteasomes (Bashore et al., 2015). In contrast, the USP domain of apo USP14 appears more dynamic and failed to interact with the ATPase ring of proteasome (Aufderheide et al., 2015). Regarding the structure of proteasome bound to USP14, the USP14-Uba1 complex binds to the periphery of the oligosaccharide-binding (OB) ring, close to Rpn1, whereas the active site of USP14 is located in the axial channel of the OB ring. Such a position allows the USP14 to efficiently cleave the polyUb chain, before the entrance of the substrate protein in the CP for degradation through the axial channel. Structural analysis showed that three surface loops, that is, BL1, BL2, and the loop between α9 and β13 of USP14, interact with the OB domains of Rpt1 and Rpt2 to anchor USP14 to proteasome. These interactions might trigger the translocation of the BL1 and BL2 loops away from the active site of USP14, leading to its activation (Huang et al., 2016). Therefore, these structural conformational changes might explain the mechanism of the opposing allosteric actions of USP14 on proteasome, that is, both the inhibitory effect of its apo form and its activating effect on proteasome upon substrate binding (Peth et al., 2009; Peth et al., 2013; Kim and Goldberg, 2017). USP14 protects substrates from degradation *via* limiting their dwell-time on the 26S proteasome. This suppression of degradation by the catalytic activity of USP14 reflects its capacity for action on a millisecond time-scale before the proteasome can initiate the degradation of the substrate (Lee et al., 2011; Collins and Goldberg, 2017). Therefore, these structural conformational changes might explain the mechanism of the opposing allosteric actions of USP14 on proteasomes, both in terms of its inhibitory effect in an apo form and its activating effect on proteasomes upon binding to substrates (Peth et al., 2009; Peth et al., 2013; Kim and Goldberg, 2017).

### Phosphorylation Promotes the Regulation of USP14 Activity

Recently emerging evidence have shown that posttranslational modification of DUBs relates to their abundance, localization, and activity (Wang and Wang, 2021b). Dysregulation of these modifications might cause various diseases, such as cancer, inflammatory, and neurodegenerative diseases (Reyes-Turcu et al., 2009; Kessler and Edelmann, 2011; Leznicki and Kulathu, 2017; Das et al., 2020). For example, phosphorylation has been reported to result in either the activation or inhibition of the activity of various DUBs through conformational changes in DUBs or DUB-substrate complexes (Hutti et al., 2009; Huang et al., 2011; Huang et al., 2012).

As USP14 is reversibly bound to and interregulated by proteasome, a fraction of USP14 might exist in a proteasome-free state (Koulich et al., 2008; Kuo and Goldberg, 2017). However, whether and by which means this autoinhibited form of USP14 serves its significant intrinsic physiological role remains unclear. As expected, USP14 is also subjected to important posttranslational modifications, such as phosphorylation. Interestingly, USP14 has been shown to have a total of seven phosphorylation sites, including Thr52, Ser143, Ser230, Thr235, Ser237, Ser302, and Ser432 (Zhou et al., 2013). Among them, the Akt-mediated USP14 phosphorylation at Ser432 activates its deubiquitinating activity and facilitates cleavage towards K48- and K63-linked chains rather than linear ubiquitin chains (Figure 5) (Xu et al., 2015).

To further explain the single amino acid phosphorylation-induced changes in the activity of USP14, a structural overlay of the free inactive form of USP14 (apo) to that of the USP14-Ubal complex was performed. It was accordingly found that the BL1 and BL2 blocking loops project over the catalytic site of USP14 and block the entrance of the ubiquitin C-terminus in the inactive form (Hu et al., 2005). Moreover, several residues of USP14, such as Phe331, Tyr333, and Ser432, were shown to sterically clash with the ubiquitin C-terminus. In particular, Ser432 is located within BL2 and its position shifts over 3–5 Å between the inactive and USP14-Ubal states (Hu et al., 2005). Therefore, phosphorylation of Ser432 might lead to considerable conformational changes in USP14. In addition, Ser432 is surrounded by a cluster of highly negatively charged residues, which electrostatically repulse the negatively charged phosphate group of the phosphorylated USP14, thus leading to the rearrangement of the BL2 loop, disturbing the inhibitory effect of BL2 on USP14 (Figure 2B) (Xu et al., 2015). Yet, elucidation of the accurate mechanism underlying the relationship between phosphorylation and deubiquitination requires the three-dimensional structure of USP14 in its phosphorylated form.

## USP14 Inhibitors

### Development and Optimization of USP14 Inhibitors

Consistent with its physiological and pathophysiological role in several important signaling pathways, USP14 has emerged as a drug target in a wide range of malignancies, including cancer and neurodegenerative diseases. However, the development of potent and selective inhibitors of USP14 remains an intrinsically attractive yet challenging task, partially owing to the highly conserved nature of DUBs (Harrigan et al., 2018).

**FIGURE 3:**
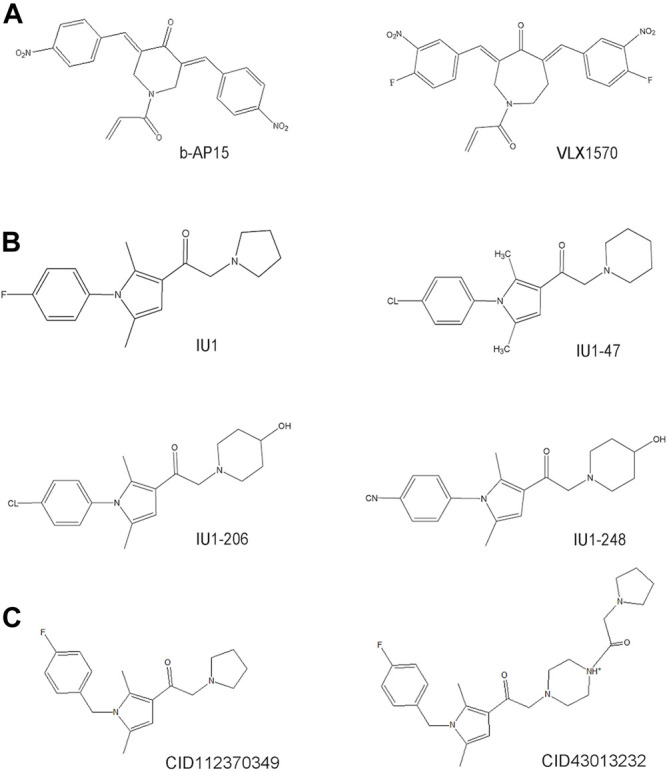
The development process and chemical structure of USP14 inhibitors. **(A)** A structure-activity relationship (SAR) study was performed based on b-AP15, and identified an analog VLX1570 with improved potency and enhanced solubility. **(B)** A high throughput screen identified the first specific inhibitors targeting on USP14, named IU1. Optimization and design of IU1 using the traditional chemistry methods allowed to the identification of IU1-47 which is 10-fold more potent specific USP14 inhibitor IU1. Based on the co-crystal structure of USP14-inhibitor complex obtained IU1-206 and IU1-248 with an IC50 value 10-fold more potent than IU1 and is comparable to the IU1-47. **(C)** Researchers employed the structural bioinformatics methods combining molecular docking identified novel potential allosteric USP14 inhibitor CID112370349 and CID43013232.

Much effort has been exerted in identifying covalent inhibitors of USP14 with poor selectivity over DUB family members. For instance, small molecule inhibitors, such as b-AP15, were identified in cell-based screening processes for compounds that could modulate the lysosomal apoptotic pathway (Figure 3) (D'Arcy et al., 2011). In particular, b-AP15 was shown to exhibit inhibitory activity on both deubiquitinases (USP14 and UCHL5) in a covalent manner. However, owing to its poor selectivity for other DUB enzymes, such as UCHL5, its therapeutic potential remains undetermined. A structure-activity relationship study based on b-AP15 was also performed. In this study, an analog (VLX1570) with improved potency and enhanced solubility (Figure 3), which was found to interact with the key catalytic cysteine residues located at the active center of both USP14 and UCHL5, was identified (Wang X. et al., 2015). Unfortunately, the clinical research on the treatment of multiple myeloma with VLX1570 was discontinued, due to severe lung toxicity. To date, several metabased compounds, including auranofin and pyrithione (PT)-metal chelates such as zinc or copper, have been found to target USP14 with promising therapeutic potential. These compounds were identified to significantly inhibit the 26S proteasome by targeting both USP14 and UCHL5 rather than the DNA in the cell, exerting safer and more potent antitumor effects (Table 1) (Zhao et al., 2016a; Chen et al., 2017; Zhao et al., 2017).

**TABLE 1:** The development process and characterization of USP14 inhibitors.

**Inhibitor**	**Development process**	**IC**_**50**_	**Selectivity**	**Cellular effect**	**References**
b-AP15	Cell-based screening	Unknown	USP14, UCHL5	Unknown	D'Arcy et al. (2011)
VLX1570	SAR-identified the analogue of b-AP15	Unknown	USP14, UCHL5	Unknown	Wang et al. (2015a)
IU1	High throughput screen using recombinant USP14	4–5 μM	USP14	Accelerate proteotoxic turnover in MEF cells; decrease the accumulation of oxidized proteins and reduce the menadione toxicity in HEK293 cells	Lee et al. (2010)
				A neuroprotective role against the ischemic stroke-induced brain injury	
				Increase the mitochondrial clearance; corrected mitochondrial dysfunction and locomotion impairment; attenuate the intrapulmonary inflammatory response	
				Promote the degradation of some cellular oncoproteins	
IU1-47	Optimization and design of IU1 using the traditional chemistry methods	0.6 μM	USP14, IsoT/USP5	Decrease tau level; accelerate the protein degradation rate and enhance phosphorylation of eIF2α	Boselli et al. (2017)
IU1-206	Optimization and design based on the co-crystal structure of USP14-inhibitor complex	Unknown	USP14	Unknown	Wang et al. (2018)
IU1-248	Optimization and design based on the co-crystal structure of USP14-inhibitor complex	0.83 μM	USP14	Unknown	Wang et al. (2018)
CID43013232	High throughput screening using structural bioinformatics methods	Unknown	USP14	Unknown	Adelakun et al. (2020)
CID 112370349	High throughput screening using structural bioinformatics methods	Unknown	USP14	Unknown	Adelakun et al. (2020)

Finley’s group was the first to report on a specific inhibitor targeting USP14 (the first inhibitor targeting DUBs), named IU1 (Figure 3) (Lee et al., 2010). A high throughput screen of 63,052 compounds based on the Ub-AMC hydrolysis assay using recombinant USP14 reconstituted with VS-26S proteasomes identified 215 USP14 inhibitors, among which only three showed selectivity for USP14. The strongest hit among these three inhibitors was IU1 with a half-maximal inhibitory concentration (IC_50_) of 4–5 μM against USP14 and good selectivity (Lee et al., 2010). Subsequently, applying traditional chemistry methods led to the design and synthesis of 87 derivatives of IU1. Using *in vivo* and *in vitro* screening assays, a 10-fold more potent and specific inhibitor of USP14 (termed IU1-47) was identified (Figure 3). IU1-47 has an IC_50_ of 0.6 μM against USP14 and shows good selectivity over the closely related IsoT/USP5 (Table 1) (Boselli et al., 2017). However, the precise molecular mechanism underlying this selectivity remains unclear as research on this compound seized. Therefore, both the toxicity and effectiveness of IU1-47 in the treatment of neurodegenerative diseases through targeting USP14 requires further investigation.

**FIGURE 4:**
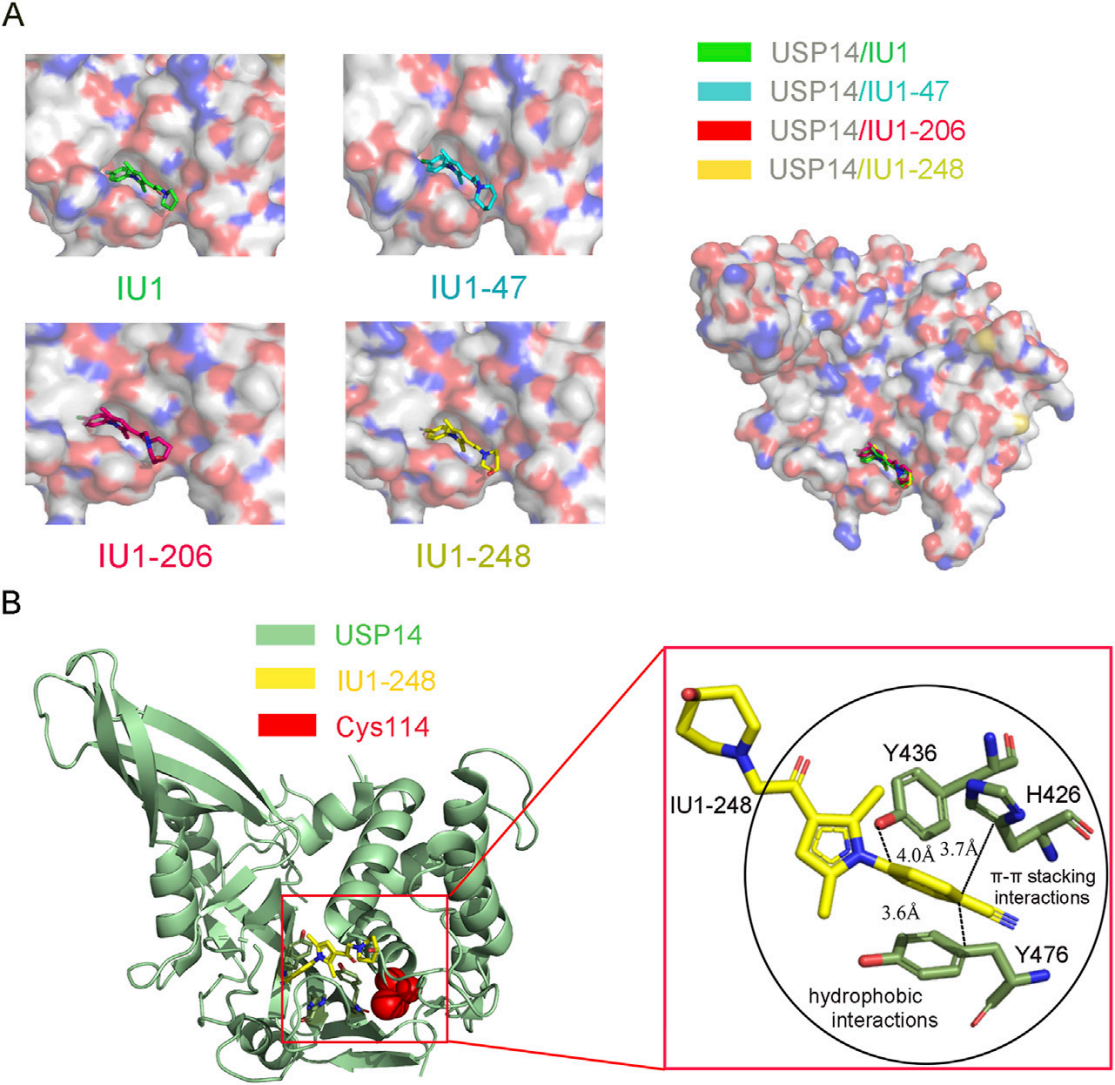
Co-crystal structure of USP14 in complex with IU1-series inhibitors. **(A)** Left: The structure of USP14 with different inhibitors. IU1, IU47, IU1-206 and IU1-248 are shown in stick model and in green, cyan, magnet and yellow, respectively. USP14 is shown in surface model and the inhibitors are shown in stick model. Right: The overall structures of USP14 with four inhibitors. **(B)** The interactions between IU1-248 and USP14. USP14, IU1-248 and Cys114 are shown in cyan, yellow and red, respectively. USP14, IU1-248 and Cys114 are shown in cartoon, stick and sphere model, respectively.

In 2018, our team reported the high-resolution crystal structure of the catalytic domain of USP14 (USP14^CAT^) bound to IU1 and IU1 derivatives (Wang et al., 2018). Structural and biochemical data pointed that IU1 and its analogs bind competitively with the ubiquitin C-terminus to the catalytic active site of USP14 through a previously unknown steric blockade mechanism. Subsequently, we performed the design and optimization of new IU1-series inhibitors, including IU1-206, based on the structural information of the interaction between IU1 and USP14 (Figure 4A). Eventually, we obtained a compound with an IC_50_ of 0.83 μM, termed IU1-248 (Figure 3), which was shown to be 10-fold more potent than IU1 and comparable to IU1-47 *in vitro* conditions (Table 1). Our study might provide valuable information for the development of USP14 inhibitors, whereas *in vivo* studies of the role of IU1-248 are lacking (Wang et al., 2018). The development of selective DUB inhibitors has been only recently pursued.

Researchers have applied structural bioinformatics techniques, combining methods such as molecular docking and molecular dynamic simulation, based on its high-resolution crystal structure to identify novel potential allosteric inhibitors of USP14 (Adelakun et al., 2020). From the screening of a library of 733 compounds with chemical structures analogous to IU1, two potential hit compounds, CID43013232 and CID 112370349 (Figure 3), were shown to have better binding affinity than IU1 (Table 1) (Adelakun et al., 2020). These results might shed light on the development of more potent and druggable USP14 inhibitors.

### Selective Inhibition Mechanism

To elucidate the molecular mechanism underlying the selective inhibition of USP14, our group determined the high-resolution crystal structure of USP14^CAT^ complexed with the IU1, IU1-47, and IU1-206 inhibitors (Figure 4A) (Wang et al., 2018). These structures indicated that IU1-series compounds share a similar binding pocket, which is 8.3 Å away from the Cys114 at the catalytic center and actin in an allosteric inhibition mode. Superimposition of USP14^CAT^-IU1 with USP14-Ubal demonstrated that IU1 inhibits USP14 by blocking the entrance of the ubiquitin C-terminus into the thumb-palm cleft where the catalytic center is buried (Figure 4A) (Wang et al., 2018).

Through systematically analyzing the detailed information of USP14^CAT^-IU1, three residues, H426, Y436, and Y476, which are conserved among most USPs, were shown to be critical for the recognition of IU1. The benzene ring of IU1 contacts with the H426, Y436, and Y476 residues *via* π–π stacking and hydrophobic interactions. In addition, the benzene ring of IU1 is perpendicular to the pyrrole ring, enforcing the interaction between the benzene ring and the H426, Y436, and Y476 residues of USP14. The two methyl groups of the pyrrole ring are also vital for the inhibitory capacity of IU1, contributing to the formation of π–π stacking (Wang et al., 2018).

Subsequently, a structure-guided design was performed to identify novel IU-series inhibitors. Considering that the dimethyl-substituted pyrrole ring and benzene ring of IU1 are essential for π–π stacking, a new inhibitor was designed and optimized based on the main backbone. To improve the solubility and binding affinity of IU1, we focused on electron-withdrawing substituents on the benzene ring critical for π–π stacking and replaced the pyrrolidine ring with other larger rings extending into the solvent-exposed region. As a result, the IU1-248 compound with significantly improved potency and solubility was developed (Wang et al., 2018).

The crystal structures of USP14^CAT^-IU1 and USP14^CAT^-IU1-248 showed that IU1-248 binds in the same pocket similar to other IU1-series inhibitors. The improved potency of IU1-248 and IU1-47 was explained based on these crystal structures. First, the Cl^−^ group of IU1-47 and CN^−^ group of IU1-248 in the phenyl ring are larger than the F^−^ atom in IU1, thereby enforcing the van der Waals interactions and sufficiently filling up the binding pocket of USP14. Second, the piperidine rings in IU1-248 and IU1-47 are larger than that in IU1, thus providing stronger hydrophobic interactions (Figure 4B). Third, molecular modeling results demonstrated that the methylene linkage between piperidine and USP14 is also critical for IU1-series inhibitors (Wang et al., 2018). Therefore, our high-resolution crystal structures allowed us to design more potent and selective USP14 inhibitors.

## Biological Functions of USP14

**FIGURE 5:**
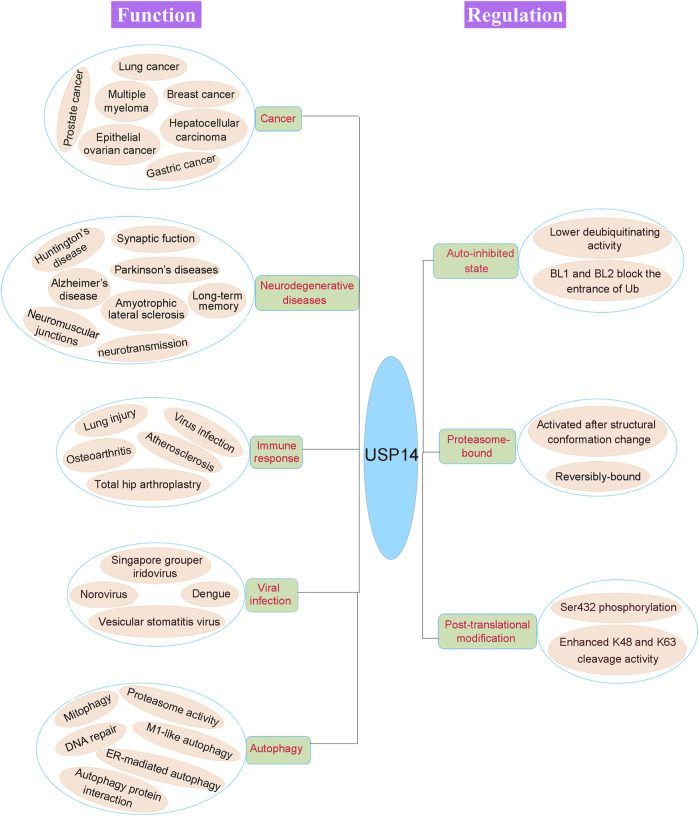
Pathophysiological function and regulation of USP14. The physiological and pathological functions of USP14 are shown in the left of the figure. Dysregulation of DUB can cause various disease, including cancer, neurodegenerative diseases, immune response, viral infection and autophagy. The activity of USP14 is strictly regulated as shown in the right of the figure. USP14 exhibited an auto-inhibited state with lower deubiquitinating activity as the auto-inhibited conformation. And the proteasome-bound USP14 gained the deubiquitinating activity. Finally, phosphorylation of USP14 at Ser432 enhanced its K48 and K63 cleavage activity.

Silencing USP14 in HeLa cells was reported to accelerate cellular protein degradation (Koulich et al., 2008). Emerging evidence indicated that USP14 manipulates disease-causing proteins through the UPS and autophagic systems. Abnormal expression of USP14 has been linked to the occurrence and progression of various tumors, neurodegenerative disorders, the immune response, and autophagy by perturbing several signaling pathways (Table 2, Figure 5) (Crimmins et al., 2006; Shinji et al., 2006; Chen et al., 2009a; D'Arcy et al., 2011; Xu et al., 2016).

**TABLE 2:** USP14 connected with various pathophysiological processes *via* different signaling pathway.

**Targeted process**	**Diseases**	**Cellular effects**	**Targeted signaling pathway**	**USP14 inhibition/knockdown**	**References**
Cancer	Breast cancer	USP14 overexpression and promote cancer cell proliferation and metastasis	Regulate cell cycle by controlling the ubiquitination level of Cyclin B1	Increase the ubiquitination of CyclinB1 and arrest cell at G2/M phase to inhibit cancer cell proliferation and migration	Fraile et al. (2012); Fortelny et al. (2014); Zhu et al. (2016a)
	ER^−^/AR^+^ breast cancer	Deubiquitination of AR and promote breast cancer growth	Regulate AR-involved Wnt/β catenin and PI3K/AKT signaling pathway	Inhibit cell proliferation and induce apoptosis by downregulating Wnt/β catenin and PI3K/AKT signaling pathway	Gucalp et al. (2013); Guerrero et al. (2013); Cochrane et al. (2014); Kono et al. (2017); Liao et al. (2018); Traina et al. (2018); Thorek et al. (2019); Xia et al. (2019)
	Multiple myeloma	Overexpression of USP14 increase cell adhesion and attenuate cell apoptosis	Regulate Wnt and Bcl-xl apoptotic signaling pathway	Downregulate Cyclin B1 and caspase-dependent apoptosis	Borodovsky et al. (2001); D'Arcy et al. (2011); D'Arcy and Linder. (2012); Damiano et al. (1999); Fortney et al. (2001); Furukawa and Kikuchi. (2015); Hazlehurst et al. (2006); Kobune et al. (2007); Rowinsky et al. (2020); Tian et al. (2014); Wang et al. (2016); Xu et al. (2017)
	Hepatocellular carcinoma (HCC)	USP14 positively regulate Wnt/β-catenin signaling by cleaving K63-linked polyubiquitin chains	Activated Wnt/β-catenin signaling in HCC patients	Alter cell cycle, suppress cell proliferation and induce cell apoptosis	Chuensumran et al. (2011); Jung et al. (2013); Huang et al. (2015); Ding et al. (2018); Wright et al. (2019); Zhang et al. (2020b)
	Epithelial ovarian cancer (EOC)	Overexpression of USP14 promote proliferation of EOC cell	USP14-BCL6 axis	Decreased BCL6 expression, slowly glow and enhanced apoptosis of EOC cell	Wang et al. (2015b); Wang et al. (2015c); Zhao et al. (2016b); Huang et al. (2017); Leeman-Neill and Bhagat. (2018); Shen et al. (2020)
	Prostate cancer (PC)	Overexpression of USP14 promote the proliferation of PC cell	Inhibit the degradation of AR through deubiquitinating the oncoprotein	Promote the ubiquitination and degradation of AR, and suppress PC cell proliferation *via* arresting that in G0/G1 phase	Way et al. (2004); Ellem and Risbridger. (2009); Singh et al. (2015); Liao et al. (2017); Ma et al. (2018); Geng et al. (2020)
	Lung cancer	Upregulated USP14 promoted tumor cell proliferation	Stabilize β-catenin	Arrested cell cycle and decreased the lung cell proliferation, migration, and invasion	Shu et al. (2011); Wu et al. (2013); Zhu et al. (2016b); Han et al. (2019)
	Gastric cancer (GC)	USP14 stabilize the oncoprotein vimentin	Akt and ERK signaling pathways	Increased sensitivity of GC cells to cisplatin by leading apoptosis through inactivating Akt and ERK signaling pathways	Zhu et al. (2017); Chen et al. (2021); Pi et al. (2021)
Neurodegenerative diseases	Alzheimer’s disease (AD)	Accumulation of the intracellular protein aggregates like tau and ataxin-3	Associate with proteasome and protect proteotoxic protein from degradation	Enhanced clearance of tau protein and ataxin-3	Lee et al. (2011); Lee et al. (2015); Ortuno et al. (2016); Boselli et al. (2017); Collins and Goldberg. (2017); Kiprowska et al. (2017)
	Parkinson’s diseases (PD)	Accumulation of intracellular protein aggregates like tau, α-synuclein, and TDP-43	Associated with proteasome and protect proteotoxic protein from degradation	Decreased tau, α-synuclein, and TDP-43	Ortuno et al. (2016)
	Amyotrophic lateral sclerosis (ALS)	Accumulation of intracellular protein aggregates like tau, α-synuclein, and TDP-43	Associated with proteasome and protect proteotoxic protein from degradation	Decreased tau, α-synuclein, and TDP-43	Ortuno et al. (2016)
	Huntington’s disease (HD)	Overexpression of USP14 reduce cellular aggregates in Htt expression cells	Associated with proteasome and protect proteotoxic protein from degradation	Overexpression of inactive USP14 have no effect on the Htt aggregates	Tsoi et al. (2012); Hyrskyluoto et al. (2014)
	Neuromuscular junctions (NMJs)	Structure of NMJ changes significantly, such as nerve terminal sprouting	Coordinate MLK3-MKK4 and JNK signaling	Loss of USP14 in axJ mice present reducing muscle mass, a resting tremor, and obvious hindlimb rigidity; Neuronal expressing inactive USP14 present motor deficits	Wilson et al. (2002); Chen et al. (2009a); Vaden et al. (2015a); Vaden et al. (2015b)
	Synaptic function	Block the maturation of NMJ	Increased GABAA receptor	Loss-of-function mutation of USP14 lead a profound effect on the neurological and synaptic defects	Chen and Sun. (2009b); Lappe-Siefke et al. (2009); Chen et al. (2011); Lee et al. (2011); Jarome and Helmstetter. (2013a)
	Defects in neurotransmission	Unable to recruit sufficient number of vesicles to match the rates of transmitter release	Catalytic-independent manner	Loss of USP14 leads a deficit of paired PPF at hippocampal synapses without changing basal release probability; overexpression of an inactive USP14 rescues PPF deficit and synaptic vesicle numbers	Wilson et al. (2002); Walters et al. (2014)
	Long-term memory	Inhibition of USP14 impaired the long-term memory through a fear conditioning task	Unknown	Impaired long-term memory	Jarome and Helmstetter, (2013a)
Immune response	Inflammatory NF-*κ*B signaling pathway	Promote I-*κ*B degradation and increase cytokine release such as TNFα, IL-8 in lung epithelial cell	Promote I-*κ*B deubiquitination	Decrease LPS-mediated TNFα and IL-6 release and ERK1/2 and IκBα phosphorylation, whereas with increased IκBα and decreased NF-κB p65 translocation from cytoplasm to nucleus	Medzhitov. (2008); Mines et al. (2009); Hayden and Ghosh. (2012); Liu et al. (2017); Wang et al. (2021a)
	Virus infection	RIG-I-induced activation of NF-κB was inhibited by USP14	Deubiquitinate K63-linked RIG-1	Promote the production of pro-inflammatory cytokines in VSV-infected macrophages or mice	Shrivastava et al. (2016); Zhong et al. (2020); Li et al. (2021)
	Lung injury	USP14 can stabilize CBP *via* removing its ubiquitination	USP14-CBP	Decrease the abundance of CBP and reduce LPS-mediated cytokine release	Wei et al. (2017)
	Osteoarthritis (OA)	Upregulated USP14 level in OA articular cartilage and chondrocytes treated with IL-1β	Activation of NF-κB pathway	Unknown	Li et al. (2019b)
	Total hip arthroplasty (THA) for osteoarthropathy	Overexpression of USP14 inhibits wear particle-induced TNFα release and NF-κB activation, as well as decreases PI3K/AKT pathway activation and macrophage polarization	USP14-NLRC5 axis by inhibiting NF-κB and PI3K/AKT pathway	Unknown	Cui et al. (2010); Massin and Achour. (2017); An et al. (2018); Zhou et al. (2018); Fang et al. (2020)
	Atherosclerosis (AS)	USP14 stabilizes and protect CD36 from degrading	Remove the ubiquitin chain on CD36	Suppress the uptake of oxLDL, subsequently decrease the formation of foam cell	Kunjathoor et al. (2002); Chistiakov et al. (2016); Zhang et al. (2020a)
Autophagy	Proteasome activity	Phosphorylation-mediated activation of USP14 negatively controlling autophagy	Deubiquitinating K63-linked Beclin1	Increased proteasome activity, and decreased cellular autophagy especially the autophagosome-lysosome fusion	Ciechanover and Kwon. (2015); Xu et al. (2016); Kim et al. (2018a)
	Mitophagy	Unknown	Unknown	Promote mitophagy in the absence of the well-studied PINK1 and Parkin and correct the locomotion behavior and mitochondrial dysfunction in PINK1/Parkin mutant *Drosophila* PD model	Chakraborty et al. (2018)
	Autophagy protein interaction	Overexpression of mutant USP14-W58A disrupt the interaction of USP14 to proteasome, while increased the binding of USP14 to HSC70 and GABARAP	USP14-HSC70 axis	By using HSC70 inhibitor, USP14-W58A promoted GABARAP autophagosome was abolished	Srinivasan et al. (2020)
	DNA repair	USP14 has higher activity in the autophagy-deficient cells	Unknown	Inhibition of USP14 can rescue the DDR defects in autophagy-deficient prostate cancer cells through directly interacted with RNF168	Sharma et al. (2018); Sharma et al. (2020)
	ER-mediated autophagy	USP14 is highly expressed in lung cancer patients and negatively regulated lung tumorigenesis through apoptosis and autophagy pathway	Activation of JNK1	Inhibition of USP14 lead the accumulation of ubiquitinated proteins that overwhelms the capacity of ER and leading ER-mediated autophagy	Han et al. (2019); Moghadami et al. (2020)
	M1-like autophagy	Neochromine S5 directly bind to USP14 and inhibits the activity of USP14	Interaction of USP14-TRAF6 and Beclin1-Bcl2	S5 dramatically inhibit the macrophage-induced inflammation by inhibiting USP14, triggering the ubiquitination of Beclin1 and autophagy	Rittirsch et al. (2009); Gao et al. (2014); Xu et al. (2020)
Viral infection	Norovirus	USP14 binds to UPR mediator IRE1, UPR is activated by ER stress which induced by the production of large amounts of viral proteins after viral infection	USP14-IRE1, ER stress-activated UPR	Infection and replication of murine or human norovirus were significantly inhibited	Kapuria et al. (2010); Perry et al. (2012)
	Dengue	Unknown	Unknown	Inhibit the replication of several flaviviruses with the most pronounced inhibition effect on dengue virus	Guzman et al. (2010); Nag and Finley. (2012)
	Vesicular stomatitis virus (VSV)	Overexpression of USP14 in Hela cells decrease RIG-I-mediated IFN-β production and increase VSV replication	Deubiquitinate the K63-linked RNA virus sensor RIG-I	Increased RIG-I-mediated IFN signaling and inhibited the replication of VSV	Gack et al. (2007); Shi et al. (2017); Li et al. (2019a)
	Singapore grouper iridovirus (SGIV)	Ectopic expression of EcUSP14 enhance the replication of SGIV and overexpression of EcUSP14 attenuated the activity of IFN-1, IFN-3 and NF-κB	IFN response and NF-κB signaling	Inhibit the SGIV replication	Huang et al. (2020)

### USP14 in Cancer

#### USP14 in Multiple Myeloma

Multiple myeloma (MM) is a malignant tumor of the blood. Despite advances in therapy over the past 2 decades, particularly the use of proteasome inhibitors, such as bortezomib and oprozomib (Chauhan et al., 2005; Gandolfi et al., 2017), drug resistance poses major limitations in the chemotherapy of MM (Damiano et al., 1999; Furukawa and Kikuchi, 2015). Overexpression of USP14 in MM cells significantly enhances cell adhesion-mediated drug resistance (CAM-DR) through the upregulation of the Wnt signaling. In addition, USP14 downregulates apoptosis by manipulating the level of expression of Bcl-xl (Xu et al., 2017). Immunostaining data have demonstrated that USP14 is overexpressed in bone marrow samples obtained from patients with MM compared with healthy donors (Figure 5). Knocking down USP14 decreased the viability of MM cells (Tian et al., 2014). Moreover, pharmacological inhibition of MM cells using the nonselective USP14 inhibitor b-AP15 induced the downregulation of cyclin B1 as well as caspase-dependent apoptosis in human MM xenograft models, preventing tumor growth and prolonging survival (Table 2) (D'Arcy et al., 2011; D'Arcy and Linder, 2012; Tian et al., 2014). Concomitantly, an optimized analogue of b-AP15, VLX1570 was found to induce the accumulation of 26S proteasome-bound polyubiquitin conjugates and the apoptotic response, thereby leading to an extended survival in MM xenograft models (Borodovsky et al., 2001; Wang et al., 2016). All these findings indicated that USP14 promotes tumorigenesis and drug resistance in MM and has been thus considered to be a candidate target for clinical trials (Table 2) (Kobune et al., 2007; Xu et al., 2017).

#### USP14 in Breast Cancer

Breast cancer is the second leading cause of cancer-related deaths among women worldwide. A growing body of evidence has suggested that USP14 contributes to the occurrence and development of breast cancer. Immunohistochemical analysis revealed the overexpression of USP14 in breast tissues compared with paired adjacent normal tissues (Figure 5) (Fraile et al., 2012; Fortelny et al., 2014; Zhu L. et al., 2016). Patients with high levels of expression of USP14 have shorter survival time than those with low levels of expression. More specifically, the increased expression of USP14 among patients with breast cancer was closely associated with poor prognosis. Moreover, cellular data demonstrated that knocking down USP14 inhibited the proliferation and metastasis of the MDA-MB-231 breast cancer cell line instead of promoting its apoptosis (Table 2) (Zhu L. et al., 2016).

Given the role of USP14 as potential target for breast cancer therapy, the molecular disease-causing mechanisms of USP14 were investigated. Liu et al. found that USP14 modulated the cell cycle progression of breast cancer cells by deubiquitinating cyclin B1, a critical regulator of the mitotic phase (M phase) (Yu et al., 2002; Porter and Donoghue, 2003; Ding et al., 2014; Liu et al., 2019). Inhibition or knockdown of USP14 significantly augmented the ubiquitination of cyclin B1 and arrested the cell cycle at the G2/M phase, thereby inhibiting the proliferation and migration of breast cancer cells, and offering the theoretical basis for the development of USP14-targeted anticancer drugs (Table 2) (Liu et al., 2019).

The androgen receptor (AR) has been demonstrated to be widely expressed in approximately 70% of patients with breast cancer. It has also been shown to promote the growth of breast cancer cells in estrogen receptor-negative (ER^−^)/AR-positive (AR^+^) patients (Gucalp et al., 2013; Cochrane et al., 2014; Kono et al., 2017; Traina et al., 2018; Thorek et al., 2019). USP14 was found to promote the UPS-mediated K48-ubiquitinated AR protein degradation. Both genetic knockdown and pharmacological inhibition of USP14 inhibits the proliferation and induces the apoptosis of AR-positive breast cancer cells (Table 2). As USP14 has been reported to be essential for breast cancer cell growth through the degradation of AR, it might be feasible to treat AR-positive breast cancer *via* the inhibition of USP14 (Liao et al., 2018).

Interestingly, combination of the enzalutamide AR antagonist with knockdown/inhibition of USP14 caused the significant downregulation of AR proteins, impeding AR-involved signaling pathways, including the Wnt/β catenin and phosphoinositide 3-kinase (PI3K)/AKT signaling pathways (Guerrero et al., 2013; Xia et al., 2019). Therefore, USP14-targeted inhibition in combination with the enzalutamide AR antagonist might represent a potential therapeutic strategy for breast cancer therapy.

#### USP14 in Hepatocellular Carcinoma

Hepatocellular carcinoma (HCC), one of the most common malignant tumors with a high morbidity rate, is the third leading cause of cancer-related deaths worldwide (Cheng et al., 2019; Tang et al., 2019).

**FIGURE 6:**
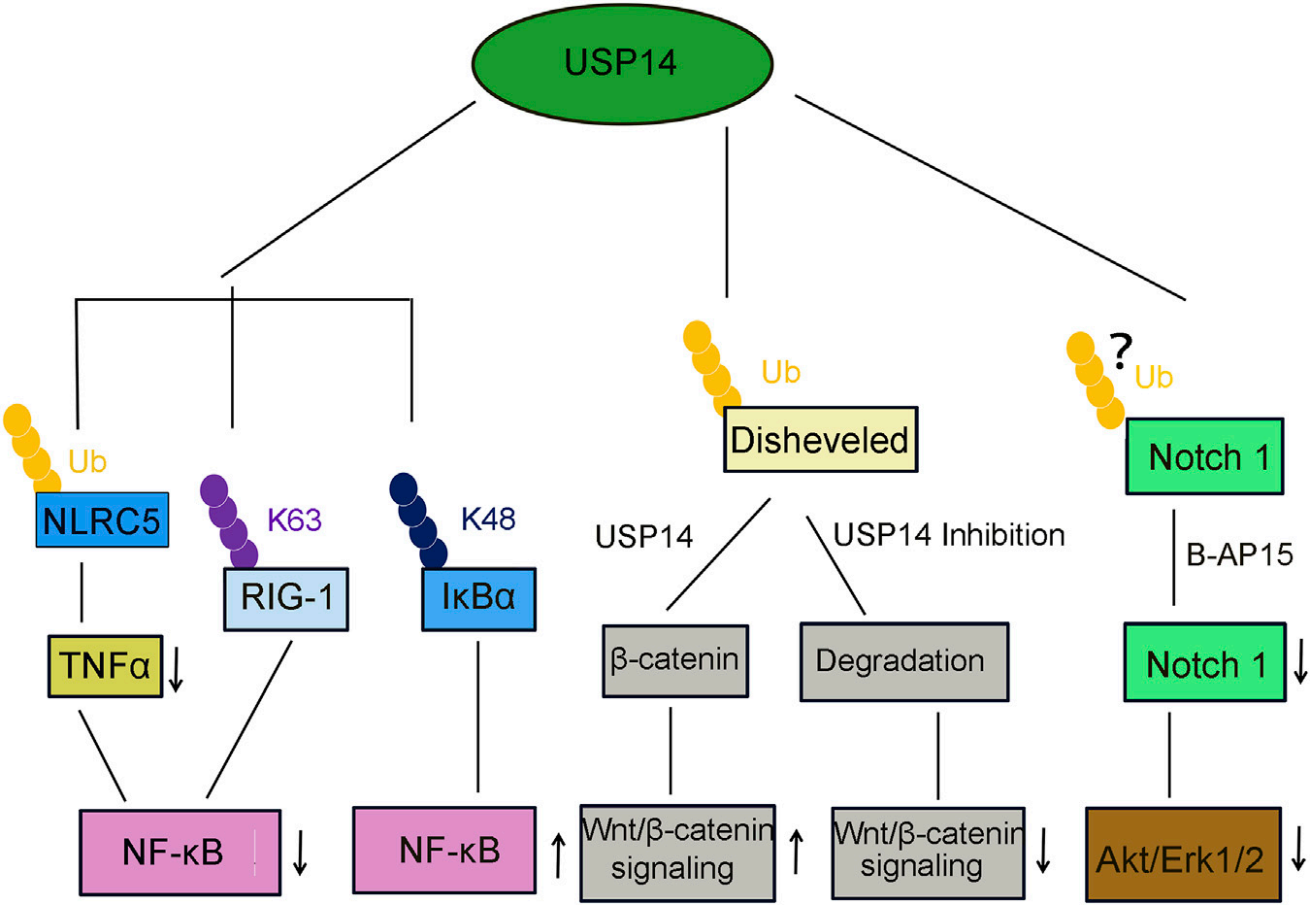
USP14 is involved in several canonical signaling pathway. USP14 deubiquitinates NLRC5, then inhibits TNFα release and NF-κB signaling activation. USP14 deubiquitinates K63-linked RIG-I and decreased the RIG-I-induced activation of NF-κB signaling. USP14 deubiquitinates K48-linked I-κBα and promote the NF-κB signaling. USP14 deubiquitinates and stabilize β-catenin and then activated the Wnt/β-catenin signaling pathway. In contrast, inhibition or knockdown of USP14 attenuated the Wnt/β-catenin signaling. Treatment of USP14 with B-AP15 suppressed the Notch1 signaling and also inhibited the phosphorylation of Akt and Erk1/2.

The level of expression of USP14 in patients with HCC is higher than that in normal cells. It has been shown to determine the differentiation of HCC cells (Figure 5) (Chuensumran et al., 2011), and has been negatively correlated with prognosis after surgery. In addition, USP14 was observed to upregulate the Wnt/β-catenin signaling mediated by cleaving K63-linked polyubiquitin chains (Table 2, Figure 6) (Jung et al., 2013) (Huang et al., 2015), and promote the proliferation and metastasis of HCC cells by deubiquitinating and activating PI3K (Table 2) (Wright et al., 2019; Zhang Y. et al., 2020). Knocking down USP14 in HCC cells was reported to alter the cell cycle and induce apoptosis.

Furthermore, pharmacological inhibition of USP14 using b-AP15 significantly diminished cell viability. Similarly, treatment with b-AP15 also stimulates the cytotoxic response of HCC cells by inhibiting the Notch1 (Figure 6) and Wnt/β-catenin signaling pathways and enhancing endoplasmic reticulum (ER) stress/unfolded protein response (UPR), thus increasing the apoptosis of HCC cells (Ding et al., 2018).

#### USP14 in Epithelial Ovarian Cancer

Epithelial ovarian cancer (EOC) is one of the three most common malignant tumors of the female genital tract, with a prevalence only less than that of cervical and endometrial cancer, but with a high mortality rate (Bowtell et al., 2015). Due to the occult progression, resistance, and difficulty in early diagnosis of EOC, most cases are discovered at the late stage, resulting in a 5-years survival rate that barely reaches 30% (Su et al., 2013; Ferrari et al., 2015). Therefore, it is highly desirable to investigate the mechanism underlying the resistance of ovarian cancer to therapy and develop more efficient therapeutic approaches for EOC.

The level of expression of USP14 is higher in EOC than that in normal ovarian tissues (Figure 5). This high level of expression of USP14 has been closely related to poor prognosis among patients with EOC (Wang Y. et al., 2015). Starvation and refeeding assays showed that USP14 exerts a critical role in the proliferation of SKOV3 cells, a representative EOC cell line. Knocking down USP14 was shown to slow down the growth and enhance the apoptosis of SKOV3 EOC cells (Wang Y. et al., 2015). In addition, platinum pyrithione (PtPT), a well-characterized synthetic chemical complex that potently inhibits the 26S proteasome-associated USP14, showed cytotoxicity towards EOC cells (Zhao et al., 2016b). PtPT accumulated ubiquitinated-protein substrates and suppressed the proliferation of EOC cells by inducing G2 phase arrest and apoptosis of SKOV3 cells. More importantly, PtPT dramatically inhibited the growth of EOC xenografts without detectable adverse effects (Huang et al., 2017). These findings implied that USP14 is a key regulator for the progression of EOC and might serve as a notable target for the treatment of EOC.

Besides, USP14 is highly expressed and confers resistance in cisplatin-sensitive EOC cells by stabilizing BCL6, a transcription factor involved in chromosomal translocation (Shen et al., 2020). Genetic knockdown or pharmacological inhibition of USP14 reversed cisplatin resistance in EOC cells and was accompanied by the decreased expression of BCL6 (Wang YQ. et al., 2015; Leeman-Neill and Bhagat, 2018). These results indicated that targeting the USP14-BCL6 axis might benefit the treatment of EOC.

#### USP14 in Prostate Cancer

Prostate cancer (PC) is the second most common cancer among men worldwide (Romero, 2018). Androgen ablation was the first effective treatment strategy used for advanced PC. However, most prostate tumors become resistant to therapy (Papandreou and Logothetis, 2004). Therefore, it is urgently required to understand the PC-causing molecular mechanism in depth and develop alternative strategies for inhibiting tumor growth. The androgen receptor (AR) is usually highly expressed and engaged in PC growth and progression (Feldman and Feldman, 2001). Overexpression of USP14, which is a novel regulator of AR (Figure 5), accelerates the proliferation of PC cells through the deubiquitination and inhibition of the degradation of AR in androgen-responsive PC cells. Consistently, genetic and pharmacological inhibition of USP14 was shown to promote the ubiquitination and degradation of AR and retard the growth of PC cells by arresting them in the G0/G1 phase (Table 2) (Liao et al., 2017). Hence, the USP14-induced enhancement in PC development through the stabilization of AR validated its potential role in PC therapy.

Furthermore, USP14 was found to upregulate the abundance and transcriptional activity of activating transcription factor 2 (ATF2), which function as both a transcription factor and oncogene in PC, *via* its deubiquitination (Ma et al., 2018), thus enhancing the proliferation of PC cells. Accordingly, siRNA-induced depletion or pharmacological inhibition of USP14 resulted in the decreased expression and inactivation of ATF2 (Geng et al., 2020). In addition, knocking down ATF2 largely attenuated the USP14-dependent proliferation of PC cells (Geng et al., 2020). This link between USP14 and ATF2 in the progression of PC demonstrated that inhibition of USP14 might represent a promising therapeutic strategy against PC. Consistently, a natural compound, apigenin, was reported to promote the apoptosis of PC cells through inhibiting the enzymatic activity of USP14 (Way et al., 2004; Singh et al., 2015).

#### USP14 in Nonsmall Cell Lung Cancer

Lung cancer, which has the highest mortality rate, is one of the leading causes of cancer-related deaths worldwide (Meador et al., 2014). Owing to late-stage diagnosis and poor treatment, the 5-years survival rate for patients with lung cancer remains very low (Wakelee et al., 2007). Therefore, there is an urgent need to obtain a comprehensive understanding of lung cancer and develop more efficacious drugs.

Upregulation of USP14 in NSCLC cells has been closely associated with increased tumor cell proliferation and the shorter overall survival of patients (Figure 5) (Wu et al., 2013). USP14 regulates lung tumorigenesis through both apoptotic and autophagic pathways (Table 2) (Zhu Y. et al., 2016; Han et al., 2019). In contrast, downregulation of USP14 arrests the cell cycle and might result in the degradation of β-catenin (Shu et al., 2011; Wu et al., 2013). Furthermore, administration of the IU1-47 small molecule or siRNA inhibition of USP14 was demonstrated to significantly decrease lung cell proliferation, migration, and invasion. Hence, USP14 plays an essential role in lung cancer and the development of USP14 inhibitors appears to be a potential therapeutic approach for lung cancer.

#### USP14 in Gastric Cancer

Gastric cancer (GC) is the third most common cause of cancer-related deaths worldwide (Bray et al., 2018). Despite recent progress in the diagnosis and treatment of GC, the overall survival of patients with GC, particularly those with late-stage disease, remains very low with a 5-years survival rate less than 30% (Ajani et al., 2017).

Vimentin is highly expressed in human GC tissues, significantly promoting the growth and migration of GC cells (Mendez et al., 2010; Zhu et al., 2017). It was also demonstrated that USP14 stabilizes vimentin *via* its deubiquitination. As expected, USP14 was found to exhibit an increased level of expression in GC tissues compared with that in matched normal tissues (Figure 5). Whereas, genetic silencing of USP14 increased the sensitivity of GC cells to cisplatin and led to cisplatin-induced apoptosis by inactivating the Akt and ERK signaling pathways (Table 2) (Zhu et al., 2017). These results revealed that USP14 could potentially serves as both a prognostic marker and therapeutic target for patients with GC.

In addition, the high expression of the *YTHDF1* oncogene in GC tissues was closely related to poor prognosis in patients with GC (Pi et al., 2020; Chen et al., 2021). Accordingly, deficiency of YTHDF1 impaired the proliferation, invasion, and tumorigenesis of GC cells. A study revealed that YTHDF1 stimulates the translation of USP14 in an N6-methyladenosine (m^6^A)-dependent manner, and reversely, USP14 upregulates the expression of YTHDF1 (Chen et al., 2021). Collectively, these results might provide useful insight into the treatment of GC.

### Link of USP14 to Diseases of the Nervous System

#### USP14 in Neurodegenerative Diseases

The accumulation of intracellular protein aggregates, such as tau in Alzheimer’s disease (AD), α-synuclein in Parkinson’s disease (PD), and TAR DNA-binding protein 43 (TDP-43) in amyotrophic lateral sclerosis (ALS) is the main pathological hallmark of several neurodegenerative diseases (Ross and Poirier, 2004; Uversky, 2007; Andersen and Al-Chalabi, 2011; Ciechanover and Kwon, 2015). Therefore, inhibiting the production of protein aggregates or enhancing their intracellular clearance seems to be a plausible strategy for preventing or slowing the progression of neurodegenerative diseases (Crosas, 2014). UPS is the main system responsible for the degradation of intracellular proteins, and as such it has been linked to the majority of neurodegenerative disorders. As a shuttling component and regulator of the 26S proteasome, USP14 might modulate the clearance of protein aggregates (Borodovsky et al., 2001; Lee et al., 2010).

The IU1 USP14 inhibitor was found to be toxic to neurons, inducing calpain-dependent tau cleavage and inhibiting mitochondrial complex I (Kiprowska et al., 2017). However, deficiency of USP14 in neurons or wt cortical neurons or siRNA silencing of USP14 in cell lines did not result in any changes in the levels of tau or ataxin-3 proteins, despite an apparent increase in the levels of phosphorylated tau (Ortuno et al., 2016; Kiprowska et al., 2017). Furthermore, specific RNA aptamers of USP14 were identified to inhibit the deubiquitinating activity of USP14 (Lee et al., 2015). Another study demonstrated that the proteasomal degradation of tau protein was enhanced in the presence of aptamers of USP14, indicating that novel USP14 inhibitory aptamers facilitated the degradation of the proteotoxic protein and protected cells from neurodegenerative toxins (Lee et al., 2015). Moreover, IU1-47, a small molecule inhibitor of USP14, was also shown to accelerate the degradation of tau protein (Table 2) (Boselli et al., 2017). The effectiveness of IU1-47 series compounds for the treatment of neurodegenerative diseases through targeting USP14 requires further investigation.

In several experiments in which USP14 was either overexpressed or knocked down using siRNA, no changes were observed in the levels of tau, α-synuclein, or TDP-43 in cells (Ortuno et al., 2016). Prion diseases are fatal neurodegenerative disorders characterized by the accumulation of the prion protein (PrP^Sc^). Treatment with IU1 was reported to reduce the levels of PrP^Sc^ in prion-infected neuronal cells, whereas overexpression of USP14 elevated the levels of PrP^Sc^ in prion-infected cells (Homma et al., 2015). In Huntington’s disease, which is caused by a CAG repeat in the huntingtin (*Htt*) gene, the accumulation of intracellular mutant Htt aggregates imposes negative effects on cellular functions (Tsoi et al., 2012). Interestingly, overexpression of USP14 was shown to reduce cellular aggregates in cells expressing mutant Htt *via* UPS degradation, whereas overexpression of inactive USP14 had little effect on Htt aggregates, indicating the involvement of USP14 in mutant Htt-induced cell toxicity (Table 2) (Hyrskyluoto et al., 2014).

#### USP14 in the Development of Neuromuscular Junctions

The neuromuscular junction (NMJ) is the synaptic structure that connects the terminal end of a motor nerve with a skeletal, smooth, or cardiac muscle (Slater, 2017). Disorders of the NMJ, where the transmission of the action potential from nerves to the attached muscle occurs, are known to cause many diseases (Hirsch, 2007).

USP14 is indispensable in the development and function of NMJs (Figure 5) (Chen et al., 2009a). For example, among ataxic (*ax* ^*J*^) mice with a spontaneously occurring mutation in USP14, the level of expression of full-length USP14 in the brain decreased to only approximately 5% (Wilson et al., 2002). Loss of USP14 in *ax* ^*J*^ mice was found to cause induction of muscle mass, resting tremor, and obvious hindlimb rigidity without detectable loss of motor neurons. However, developmental defects were notable in motor neuron endplates and the structure of the NMJ was found to be significantly altered, as evidenced by the accumulation of phosphorylated neurofilaments and nerve terminal sprouting (Chen et al., 2009a). In addition, the neuronal expression of inactive USP14 has been linked to motor deficits and altered NMJ structure. Specifically, the activation of mixed lineage kinase 3 (MLK3), the downstream target MAP kinase 4 (MKK4), and c-Jun N-terminal kinase (JNK) was also observed (Table 2). Therefore, USP14 was assumed to coordinate intracellular signaling pathway to regulate the development and functions of NMJ (Vaden et al., 2015a; Vaden et al., 2015b).

#### USP14 in Synaptic Function and Transmission

Synapses are plastic and highly dynamic during development for remodeling of the protein composition (Yi and Ehlers, 2007). Consistent with the findings that protein synthesis and turnover are key to synaptic function, alternations in protein turnover and synaptic function contribute to the development of a wide range of neurological diseases (Hegde and Upadhya, 2007; Lee et al., 2011).

UPS is considered to be involved in restricting the control of protein stability required for the function of the synapse (Lee et al., 2011). In addition, USP14 has been shown to have an essential role in the maintenance of synaptic function. For instance, *ax* ^*J*^ mice showed increased levels of the gamma-aminobutyric acid (GABA_A_) receptor in the Purkinje cell membrane, and an interaction was also identified between USP14 and the GABA_A_ α1 receptor subunit, providing molecular insights into understanding the presentin disorder in *ax* ^*J*^ mice (Wilson et al., 2002; Lappe-Siefke et al., 2009).

Loss-of-function mutation of USP14 leads to a profound effect on neurological and synaptic defects (Figure 5), such as developmental disorders resulting from the disruption of NMJ maturation (Chen and Sun, 2009b). However, transgenic overexpression of ubiquitin in neurons of *ax* ^*J*^ mice was shown to prevent early postnatal lethality, restore muscle mass, and correct deficits resulting from the loss-of-function of USP14 (Chen et al., 2011). These results suggested the reason for the dramatic disorder in *ax* ^*J*^ mice following the loss of USP14 and further demonstrated that USP14 is required for maintaining ubiquitin homeostasis during synaptic development and function (Chen et al., 2011).

Defects in neurotransmission observed in the nervous system of *ax* ^*J*^ mice were proposed to make nerve terminals unable to recruit enough vesicles to match the rates of transmitter release (Bhattacharyya et al., 2012). Therefore, ubiquitination of synaptic proteins might determine the size of synaptic vesicles and release of neurotransmitters. A previous study revealed that loss of USP14 led to a deficit in paired-pulse facilitation (PPF) at hippocampal synapses without changing basal release (Wilson et al., 2002). In addition, the number of synaptic vesicles was significantly reduced, whereas overexpression of inactive USP14 rescued these effects. In conclusion, USP14 affects the synaptic structure and plasticity in a catalytic-independent manner (Walters et al., 2014). Furthermore, pharmacological inhibition of USP14 using IU1 was reported to impair long-term memory through fear conditioning, indicating a fundamental role of USP14 in long-term memory formation (Table 2) (Jarome et al., 2013b). However, the utilization of USP14 as a drug target of choice in memory formation needs to be further validated.

### USP14 in Immune Response

#### USP14 in the Inflammatory NF-*κ*B Signaling Pathway

Various studies have demonstrated the central role of USP14 in immune response *via* the regulation of the turnover of key signaling molecules implicated in immune pathways (Wang et al., 2021a). For example, the CXCR4 chemokine receptor is essential to the immune system and its dysregulation leads to an inflammatory response (Mines et al., 2009). As a result to stimulation with the CXCL12 CXCR4 agonist, CXCR4 was found to be colocalized and interacting with USP14. Moreover, knocking down USP14 stablized CXCR4, whereas its overexpression downregulated the levels of CXCR4 (Mines et al., 2009).

Inflammation triggers a series of reactions (Medzhitov, 2008), particularly the persistent activation of the NF-*κ*B signaling pathway (Hayden and Ghosh, 2012) in response to biological, physical, and chemical harmful external stimuli. For instance, overexpression of USP14 was found to promote the phosphorylation and subsequent ubiquitination of I-*κ*B, which is then submitted to the proteasome for degradation, releasing cytokines, such as tumor necrosis factor-α (TNFα) and interleukin 8 (IL-8) in lung epithelial cells (Liu et al., 2017). In addition, treatment with lipopolysaccharide (LPS) induced the serine phosphorylation of USP14, further deregulating the levels of I-κB in USP14-overexpressing cells (Table 2) (Liu et al., 2017). Together, these data might provide new therapeutic insights into the treatment of inflammatory lung diseases.

Furthermore, knocking down or pharmacological inhibition of USP14 caused the diminished LPS-mediated release of TNFα and IL-6 as well as deregulated the levels of phosphorylated ERK1/2 and IκBα, upregulated the levels of the NF-κB inhibitor IκBα, and reduced the translocation of NF-κB p65 from the cytoplasm to the nucleus (Liu et al., 2017). These results implied that USP14 promotes the activity of NF-κB and the phosphorylation of ERK1/2 induced by microbial infections (Table 2).

Viral infections stimulate the innate immune response, including the inflammatory response, which is a crucial biochemical reaction against the invasion by microbial pathogens (Figure 5) (Shrivastava et al., 2016). Retinoic acid-inducible gene I (RIG-I) is an essential recognition receptor of the innate immune system that detects RNA viruses (Zhong et al., 2020). Genetic knockdown or pharmacological inhibition of USP14 was demonstrated to significantly promote the production of proinflammatory cytokines in macrophages of mice infected by vesicular stomatitis virus (VSV). Moreover, USP14 also impeded the activation of NF-κB through deubiquitinating the K63-linked RIG-1, implying that USP14 plays a negative regulatory role in the RIG-I-triggered inflammatory response (Table 2, Figure 6) (Li et al., 2021).

#### USP14 in Inflammatory Diseases

Dysregulation of USP14-involved inflammatory signaling pathways can lead to various inflammatory diseases, such as lung injury. The ubiquitination-dependent degradation of CREB-binding protein (CBP), which is crucial for the expression of cytokine-encoding genes, was shown to reduce the LPS-induced cytokine release in mouse lung epithelial cells (Wang et al., 2010). USP14 stabilizes CBP by diminishing its ubiquitination, whereas inhibiting USP14 deregulates the abundance of CBP and prevents LPS-mediated cytokine release. These findings suggested that USP14 plays an important role in lung inflammation (Wei et al., 2017).

Osteoarthritis (OA) is an inflammatory joint disease (Ioan-Facsinay and Kloppenburg, 2017). A previous study found that the levels of USP14 were upregulated in an NF-κB pathway-dependent manner in the articular cartilage in OA and in chondrocytes treated with IL-1β (Figure 5) (Li M. et al., 2019). In turn, USP14 enhanced the activation of NF-κB by inducing the degradation of IκBα (Figure 6). Finally, USP14 promoted the dedifferentiation effect of IL-1β on chondrocytes, whereas inhibition of NF-κB remarkably reversed this effect, implying that NF-κB efficiently regulates the function of USP14 (Li M. et al., 2019). Overall, a feed-forward loop driven by NF-κB and USP14 in promoting the effect of IL-1β on chondrocytes might offer potential hints on therapeutic interventions for OA (Table 2).

Total hip arthroplasty (THA) is a highly successful and widely-applied surgical intervention for the treatment of patients with end-stage degenerative and inflammatory osteoarthropathy (Man et al., 2017). However, wear particles generated from the friction of tough bearing artificial joints might limit the long-term survival of THA, owing to risks of initiating inflammatory reactions and periprosthetic osteolysis (Massin and Achour, 2017). Therefore, it is essential to develop new strategies to attenuate wear particle-induced periprosthetic osteolysis, as well as prolong the longevity of artificial joints. NF-κB and PI3K-AKT Ser/Thr kinase (AKT) signaling pathways contribute to wear particle-induced osteolysis (An et al., 2018; Zhou et al., 2018). Moreover, USP14 has been identified to cleave the ubiquitin chains from NLRC5 (Cui et al., 2010) and inhibit the activation of the PI3K/AKT signaling and macrophage polarization, decreasing the wear particle-induced release of proinflammatory TNFα and activation of NF-κB (Table 2, Figure 6). The USP14-NLRC5 axis was found to suppress titanium wear particle-induced osteolysis by inhibiting the NF-κB and PI3K/AKT pathways, presumably offering new insights into improving the long-term survival of THA (Figure 5) (Fang et al., 2020).

Atherosclerosis (AS), a chronic and progressive inflammatory disease, is associated with heart disease, peripheral artery disease, and stroke (Writing Group et al., 2016; Moore and Tabas, 2011). Recently, it was reported that the formation of foam cells is crucial for the pathogenesis of AS (Yuan et al., 2012). Under conditions of hypertension and hyperlipidemia, low-density lipoprotein (LDL) particles are deposited and oxidized into oxLDL in the subendothelial membrane. When stimulated by these strong inflammatory factors, monocytes are activated and transform into macrophages, which then ingest modified LDL particles including oxLDL, resulting in the accumulation of cholesterol lipids and the formation of foam cells through scavenger receptors such as CD36 (Kunjathoor et al., 2002; Chistiakov et al., 2016). Inhibition of USP14 was shown to remarkably block the uptake of oxLDL, subsequently impeding the formation of foam cells. Furthermore, USP14 stabilizes and rescues CD36 from degradation by removing the ubiquitin chain. Interestingly, blocking CD36 with antibodies significantly attenuated the effect of USP14 on the formation of foam cells (Table 2) (Zhang F. et al., 2020). Therefore, inhibition of USP14 can decrease the formation of foam cells by downregulating the CD36-mediated uptake of lipids, hence providing novel insights into AS-targeted interventions (Figure 5).

### USP14 in Autophagy

#### USP14 Links UPS to Autophagy

UPS and autophagy are the two main degradative pathways that function complementarily to strictly monitor the turnover of intracellular proteomes in eukaryotes. UPS is mainly responsible for degrading short-lived proteins conjugated with K48-linked ubiquitin chains under normal conditions (Rock et al., 1994; Komander and Rape, 2012; Kocaturk and Gozuacik, 2018; Wang et al., 2020). Conversely, autophagy is mainly in charge of the clearance of long-lived proteins and intracellular organelles encapsulated in autophagosomes under starvation or stressful conditions (Klionsky, 2007; Kocaturk and Gozuacik, 2018; Wang et al., 2020).

Given their nature of degrading misfolded proteins, the simultaneous activation of UPS and autophagic system might presumably provide an efficient strategy for the clearance of misfolded proteins under pathological conditions, such as neurodegenerative diseases (Ciechanover and Kwon., 2015). However, the mechanism underlying the common regulation of UPS and autophagy remains unclear. USP14 has been demonstrated to regulate autophagy by deubiquitinating K63-linked Beclin, analogous to its inhibitory role in UPS *via* the trimming of K48 ubiquitin chains (Xu et al., 2016). A recent study showed that Akt-induced phosphorylation-mediated activation enhanced the activity of USP14 for cleaving K63 ubiquitin chains, indirectly negatively regulating autophagy (Xu et al., 2016). Furthermore, inhibition of USP14 enhanced the activity of the 26S proteasome, while significantly decreased cellular autophagy, especially the autophagosome-lysosome fusion, which constituted compensatory negative feedback between UPS and autophagy (Table 2) (Kim et al., 2018a).

Mitophagy is an autophagic response that selectively degrades dysfunctional mitochondria to avoid the production of excessive reactive oxygen species and activate cell death (Chan et al., 2011; Chen et al., 2013). Both pharmacological inhibition and genetic knockdown of USP14 were reported to promote mitophagy in the absence of PINK1 and Parkin (Figure 5) (Chakraborty et al., 2018). Inhibition of USP14 also corrected the locomotive behavior and mitochondrial dysfunction in a *PINK1*/*Parkin* mutant *Drosophila* model of PD (Table 2) (Chakraborty et al., 2018). These studies provided a novel intervening target for alleviating mitochondrial dysfunction and PD-coupled symptoms.

Moreover, the dynamic interaction of the HSC70 chaperone with USP14 is known to mediate the crosstalk between proteasome and autophagy. Overexpression of mutant USP14-W58A disrupted the interaction of USP14 with the 26S proteasome, while enhanced the binding of USP14 to the HSC70 chaperone and GABARAP autophagic protein (Srinivasan et al., 2020). In striatal mutant huntingtin-expressing neurons with reduced expression of USP14 and HSC70, overexpression of USP14-W58A promoted the formation of the GABARAP autophagosome, which was conversely abolished following the application of an HSC70 inhibitor (Table 2) (Srinivasan et al., 2020). These findings demonstrated that modulating the USP14-HSC70 axis might offer an effective target in the management of Huntington’s disease by disturbing multiple proteastatic pathways.

#### USP14-Mediated Autophagy in DNA Repair

Ionizing radiation (IR) is a highly effective modality for the treatment of solid tumors, including PC (Begg et al., 2011). IR-induced lesions of DNA double-strand breaks (DSBs) are recognized and repaired by the DNA damage response (DDR) signaling pathway. More specifically, non-homologous end joining (NHEJ) is the major DSB repair pathway in response to IR (Table 2) (Escribano-Diaz et al., 2013). However, resistance of tumor cells to IR poses limitations to the effectiveness of this modality.

Recent studies demonstrated that although USP14 had higher activity in autophagy-deficient cells, the ring finger protein 168 (RNF168, a E3 ubiquitin ligase)-related ubiquitin signaling and the formation of tumor protein p53 binding protein 1 (TP53BP1) IR-induced foci (IRIF) were significantly decreased (Sharma et al., 2018). However, inhibition of USP14 rescues the DDR defects in autophagy-deficient PC cells (Sharma et al., 2018). In addition, chromatin recruitment by critical NHEJ proteins, such as Ku70 was found to be diminished in autophagy-deficient cells (Sharma et al., 2020). Interestingly, USP14 deubiquitinates and interacts with core NHEJ proteins, whereas its inhibition rescues the activity of NHEJ-DDR proteins. Similar to the inhibition of USP14, blocking AKT, which mediates the phosphorylation of USP14, was also reported to rescue the activity of NHEJ-DDR proteins during autophagy in PTEN-deficient cells (Sharma et al., 2020). These findings provided a unique mechanism underlying the connections among USP14-mediated autophagy, DDR-related ubiquitin signaling, and DNA repair (Figure 5).

#### USP14-Mediated Autophagy in Lung Cancer

Nonsmall cell lung cancer accounts for more than 80% of cases of lung cancer, which is the leading cause of cancer-related deaths worldwide (Goldstraw et al., 2011; Minguet et al., 2016). USP14 is highly expressed in patients with lung cancer. Genetic or pharmaceutical inhibition of USP14 has been shown to significantly decrease the proliferation, migration, and invasion of lung cancer cells. Notably, USP14 negatively regulates lung tumorigenesis *via* both the apoptotic and autophagic pathways (Han et al., 2019). Furthermore, inhibition of USP14 led to the accumulation of ubiquitinated proteins, overwhelming the capacity of the ER and triggering ER-mediated autophagy. In A549 lung cancer cells, inhibition of USP14 induced ER stress-mediated autophagy by activating JNK1 (Table 2) (Moghadami et al., 2020). These findings indicated a new mechanism by which inhibition of USP14 causes ER stress-mediated autophagy in A549 lung cancer cells (Figure 5).

#### USP14-Mediated Autophagy in Inflammation

Inflammation is a normal biological response to harmful stimuli, including pathogens, irradiation, and damaged cells (Thaiss et al., 2016). However, excessive stimulation of the inflammatory response leads to inflammatory diseases, such as sepsis (Takeuchi and Akira, 2010). Macrophages, including M1-like and M2-like macrophages, are essential components of the innate immune system, and play critical roles in the inflammatory response and host defense (Mantovani et al., 2002; Gordon and Martinez, 2010). Treatment with the neochromine S5 compound was shown to significantly elevate autophagy in the form of M1-like autophagy, as S5 was directly bound to USP14 at Ser404, Phe405, and Cys414 through hydrogen bonds, inhibiting the activity of USP14 (Gao et al., 2014; Xu et al., 2020). This complex was found to block the interaction of USP14 with TNF receptor associated factor 6 (TRAF6), subsequently promoting the ubiquitination of Beclin 1 by disturbing the Beclin 1-Bcl2 interaction (Table 2). As a result, accumulation of autophagosomes was observed in macrophages, finally inducing the blockade of M1-like macrophage polarization and alleviating cecal ligation and puncture (CLP)-induced sepsis (Rittirsch et al., 2009). In summary, S5 dramatically inhibited macrophage-induced inflammation by counteracting USP14, triggering the ubiquitination of Beclin 1 and autophagy (Figure 5) (Xu et al., 2020).

### USP14 in Viral Infection

UPS is crucial in a majority of cellular processes, including the response to viral infections. To overcome the resistance host to viral invasion and propagation by UPS, viruses have evolved the capability to sophisticatedly reprogram UPS, rendering the cellular environment favorable to viral replication (Nag and Finley, 2012). In a proteomics study, the WP1130 nonselective small molecule inhibitor was identified to inhibit the replication of murine or human norovirus through targeting USP14 (Kapuria et al., 2010). Genetic knockdown or chemical inhibition of USP14 also interfered with norovirus infection (Figure 5) (Perry et al., 2012). Moreover, USP14 has been shown to bind to the inositol-requiring enzyme, a mediator of the UPR, which is activated by ER stress induced by the production of large amounts of viral proteins after viral infection. In addition, WP1130 inhibition or UPR induction also inhibited the occurrence of infections by other RNA viruses, including the Sindbis virus, encephalomyocarditis virus, and La Crosse virus (Table 2) (Perry et al., 2012). Therefore, targeting DUBs or the UPR is promising for the development of antiviral therapies.

Dengue, caused by a virus belonging to the flavivirus family, is one of the most common infectious diseases worldwide (Guzman et al., 2010). The IU1 USP14 inhibitor inhibits the replication of several flaviviruses, exhibiting its most pronounced inhibitory effect on the dengue virus (Figure 5) (Nag and Finley, 2012). These findings provided new interventional targets for the treatment of infections caused by the dengue virus and other flaviviruses.

USP14 negatively regulates the initiation of antiviral responses by directly deubiquitinating the K63-linked RNA virus sensor, RIG-I (Gack et al., 2007; Shi et al., 2017). Either knocking down or inhibiting USP14 using IU1 was shown to significantly increase the RIG-I-mediated interferon (IFN) signaling and inhibit the replication of VSV (Figure 5) (Li H. et al., 2019). In contrast, overexpression of USP14 in HeLa cells deregulated the RIG-I-mediated expression of IFN-β and led to elevated VSV replication (Table 2) (Li H. et al., 2019). These findings provided new insights into the treatment of RNA viral infections by targeting USP14.

Interestingly, a USP14 homolog isolated from the orange spotted grouper (EcUSP14) has been demonstrated to play a similar role in viral infections in fish (Huang et al., 2020). Ectopic expression of EcUSP14 dramatically enhanced the replication of Singapore grouper iridovirus (SGIV), whereas treatment with IU1 significantly suppressed SGIV replication (Huang et al., 2020). In addition, overexpression of EcUSP14 attenuated the activity of IFN-1, IFN-3, and NF-κB (Huang et al., 2020). Therefore, EcUSP14 was assumed to promote the replication of SGIV by negatively regulating the IFN response to viral infections.

## Conclusion and Future Perspectives

Over the past few years, USP14 has emerged as a potential drug target for a wide range of diseases owing to its pivotal roles in canonical signaling pathways through the modulation of protein homeostasis. Wild type USP14 exists in an autoinhibitory state with the BL1 and BL2 surface loops in the palm subdomain blocking the entrance of substrates. Activation of USP14 is tightly regulated through either 26S proteasome-bound activation or phosphorylation to ensure its biological function. Structural evidence has shown that binding to the 26S proteasome unlocks the autoinhibitory conformation of USP14. However, the mechanism by which phosphorylation activates USP14 remains elusive. Based on the structure of USP14, several inhibitors with high potency and selectivity, such as IU1 and IU1 derivatives, have been discovered and characterized for their ability to inhibit 26S proteasome-activated USP14 in an allosteric manner at both cellular and *in vivo* levels. In theory, this has enabled the design of more potent and specific inhibitors targeting USP14 and other DUBs. However, the poor inhibitory efficiency of IU1 derivatives has impeded drug development for targeting USP14. Moreover, no selective inhibitors towards phosphorylated USP14 have been identified to date. These preclinical efforts have provided some important hints and could benefit therapeutic interventions in the future by advancing the early clinical development of selective inhibitors with high potency towards USP14.

## Abbreviations

USP14, Ubiquitin-specific protease 14; DUB, Deubiquitinating enzyme; NF-κB, Nuclear factor kappa B; M phase, Mitotic phase; PI3K, Phosphoinositide 3-kinase; HCC, Hepatocellular carcinoma; PtPT, Platinum pyrithione; ATF2, Activating transcription factor 2; NMJ, Neuromuscular junction; GABAA, Gamma-aminobutyric acid; TNFα, Tumor necrosis factor-α; I-κB, Inhibitor of NF-κB; RIG-I, Retinoic acid-inducible gene I; IL-8, Interleukin 8; LDL, Low-density lipoprotein; IFN, RIG-I-mediated interferon; OB ring, Oligosaccharide-binding ring.
